# Ethical issues in neurosurgery – A scoping review from the EANS Ethico-legal committee

**DOI:** 10.1016/j.bas.2026.105946

**Published:** 2026-01-19

**Authors:** Emilia Westarp, Tim Jonas Hallenberger, Massimiliano Visocchi, Nikolaos Syrmos, Marike L.D. Broekman, Nicolàs Samprón, Kayode Agboola, Ulrika Sandvik, Naci Balak, Ciaran Bolger, Tiit Mathiesen, Mario Ganau, Jehuda Soleman

**Affiliations:** a Department of Neurosurgery, University Hospital Basel, Basel, Switzerland; b Chair Research Centre, Master II Degree and Operative Unit of CVJ Neurosurgery, Institute of Neurosurgery, Catholic University of Rome, Italy; c Department of Clinical Medicine, University of Copenhagen, Department of Neurosurgery, University Hospital of Copenhagen, Copenhagen, Denmark; d Department of Clinical Neuroscience, Karolinska Institute, Stockholm, Sweden; e Aristotle University of Thessaloniki, Greece; f Department of Neurosurgery Leiden University Medical Center and Haaglanden Medical Center, Leiden, the Netherlands; g Servicio de Neurocirugia, Hospital Universitario Donostia, San Sebastián, Spain; h Institute of Neurosurgery, Kiev, Ukraine; i Nuffield Department of Clinical Neurosciences, University of Oxford, UK; j Department of Neurosurgery, Karolinska University Hospital, Stockholm, Sweden and Department of Clinical Neurosciences, Division of Neurosurgery, Karolinska Institute, Stockholm, Sweden; k Department of Neurosurgery, Istanbul Medeniyet University, Istanbul, Türkiye; l Department of Clinical Neuroscience, RCSI, Dublin, Ireland; m BAU International University Batumi, Batumi, Georgia

**Keywords:** Ethical issues, Ethicolegal, Guidelines, EANS, Education

## Abstract

**Background:**

Ethical considerations are integral to neurosurgical decision-making, yet emerging technologies, demographic shifts, and global crises continuously introduce new challenges. Key ethical concerns include patient autonomy, prioritization, the value of life, research ethics, and personality-altering procedures. Despite their importance, formal ethics training for neurosurgical residents is often lacking, and guideline application remains inconsistent. This scoping review summarizes current literature on ethical issues in neurosurgery, identifies key topics and assessment methods, and highlights research priorities to enhance ethical awareness.

**Methods:**

A systematic literature search was conducted in Medline, Embase, and Web of Science using the search strategy (Ethic∗[Title]) AND (neurosurg∗[Title]). The search, performed on October 8, 2024, yielded 334 records (1985–2024). After removing duplicates and screening, 13 studies met inclusion criteria. Two independent reviewers selected original research in English or German addressing ethical neurosurgical issues, excluding opinion pieces, reviews, and case reports. Extracted data included study characteristics, country, year, topic, design, and key findings.

**Results:**

From a neurosurgical perspective, six ethical subcategories emerged: decision-making (31 %), pediatric neurosurgery (23 %), neurosurgery in developing countries (15 %), artificial intelligence (15 %), functional neurosurgery (8 %), and patient care (8 %). From a classical ethical standpoint, seven studies (53.8 %) focused on psychosocial ethical issues, four (30.7 %) examined normative ethical questions, and two (15.4 %) addressed aspects of professional ethics. All studies employed a qualitative research design. Most studies (77 %) used questionnaires or structured interviews for data collection. Findings revealed regional differences in ethical decision-making, increasing reliance on hospital ethics committees, resource-related dilemmas in low-income countries, and emerging AI-related concerns. Despite growing interest, structured assessment methods and standardized ethics education remain limited.

**Conclusions:**

Ethical challenges in neurosurgery, as explored through the lenses of indirect sources (published literature), are diverse and shaped by technological advancements and sociopolitical factors. AI-related ethics and crisis-driven dilemmas, such as those arising from wars and pandemics, are gaining attention. However, research methodologies remain inconsistent, limiting data comparability. Future studies should focus on enhancing ethics training and developing standardized frameworks for ethical analysis improving neurosurgical ethical decision-making.

## Highlights

• 13 studies between 1985 and 2024 on ethical issues in neurosurgery included.
• 6 subcategories: decision making, pediatric and functional neurosurgery, developing countries, artificial intelligence and patient care.
• 77 % used questionnaires or structured interviews for data collection.
• Regional differences in ethical decision making and emerging AI related concerns.
• Limited assessment methods, ethical education and knowledge of the EANS guidelines.

## 1 Introduction

For our care to respect patients and to be in their best interest, ethical considerations, should be the cornerstone of all neurosurgical decisions. This said, clarity is needed regarding the meaning of the term “ethics”, which is often used as an umbrella expression for the philosophical study of morality, encompassing all moral principles, and values, which are supposed to guide the human conduct. Applied ethics and meta-ethics are distinct branches of ethics: applied ethics uses ethical principles to solve specific, real world dilemmas, like withdrawal of care, while meta-ethics investigates the fundamental nature of morality itself, asking questions like: what make us humans or what is worth living for?

Such preliminary distinction set the stage to explore what real world dilemmas matter the most for practicing neurosurgeons, and for this reason it is worth clarifying that the three main pillars of applied ethics consist in “normative”, “professional” and “sociological” ethics (Beauchamp et al., 2019; Banks, 2012; Durkheim, 2018)

Normative ethics provide considerations on what is morally right or wrong, with the goal to define standards and guide moral decision-making. Professional ethics applies to the preparation of standard operating procedures, national and international guidelines, codes of conduct and code of ethics within a professional context, with the goal to ensure accountability and trust in professional rules. Sociological ethics examines social factors that influence ethical beliefs and practices, looking at ethical behaviors protecting human subjects from harm and ensuring their rights, including privacy and informed consent. Furthermore, the goal of sociological ethics is also to understand how ethical norms operate in practice across cultures and even between different micro-cultures within homogenous populations.

Within this framework, applied ethical analysis benefits from a clearer articulation of the theoretical lenses through which moral problems are interpreted. While the three pillars outlined above define *where* ethical considerations operate in clinical practice, ethical theories help explain *how* moral judgments are formed and justified. Clarifying these foundational concepts allows for a more precise discussion of ethical reasoning as it appears in neurosurgical decision-making and in the literature analysed in this study. Deontology is an ethical theory that focuses on rules and holds that an action is morally right if it conforms to established moral principles, regardless of its outcome (Beauchamp et al., 2019). Consequentialism evaluates actions based on their outcomes or consequences, such that an action is morally right if it produces the best overall results (Hulsbergen et al., 2020). Virtue ethics, by contrast, emphasizes the character and moral qualities of the individual acting, rather than adherence to rules or the evaluation of outcomes (Asif et al., 2024).

Over the years, emerging technologies, shifts in age distribution, and recently pandemics, have confronted the neurosurgical community with new ethical challenges (Hulsbergen et al., 2020; Asif et al., 2024; Machine learning risk prediction of, 2021; Rubulotta et al., 2020; Ridwan et al., 2021).

Additionally, considerations like cultural background, religion, ethnicity, and family background play a key role and introduce a great variety of factors that need to be considered. Ethical decisions for neurosurgeons in the western world include mostly end-of life decisions in elderly or critically ill patients, as well as emergency situations, such as offering decompressive hemicraniectomy surgery in patients with bilateral fixed pupil (Tian et al., 2021). Nevertheless, exceptional circumstances like the COVID-19 pandemic, wars and the resulting refugee crisis show the need for constant re-consideration of ethical and moral standards (Ozisik et al., 2022; Death following pulmonary complications of, 2021; Ganau et al., 2020).

Not only do medical emergencies pose ethical challenges, depending on the circumstances, elective surgeries, benign lesions, and neurosurgical procedures for psychiatric disorders can require ethical consideration (Steyn et al., 2021; Lipsman et al., 2010). Ethical guidelines, education in ethics and knowledge of ethical theories can support professionalism during complex decision making (Tewarie et al., 2020; Sobhani et al., 2016). To ensure continuous high standards and patient safety in high-risk and high-cost specialties like neurosurgery, ethical knowledge and education are essential (Balak et al., 2022). Nevertheless, very few residents receive extracurricular training in medical ethics and a survey revealed that only a minority of European neurosurgeons are familiar with and apply neurosurgical ethical guidelines (Sobhani et al., 2016; Mathiesen et al., 2022).

In this scoping review led by the EANS Ethico-Legal Committee we summarize the knowledge and literature that has been gained regarding ethical issues in Neurosurgery. The aim is, to better understand what kind of ethical topics have already been evaluated, which topics are considered ethical challenges, and which methods were used for their assessment within the neurosurgical community. Consequently, the awareness for ethical considerations in neurosurgery can be raised and future research priorities defined.

## 2 Methods

### 2.1 Literature search

The study protocol was registered at Open science Framework (registration DOI 10.17605/OSF.IO/5RS9E). We established a search-strategy for the following MeSH (Medical Subject Headings) terms: Ethic∗[Title] AND neurosurg∗[Title], as well as searching for variations of the terms “ethic” and “neurosurgery” in the titles of the respective works across three different databases (Medline, Embase and Web of Science, full search string in the supplemental materials). The search was conducted on the 8th of October 2024 and included studies without limitation to the publication year.

For management of citations and identification of duplicates, EndNote (version X9.3.3, Thomson Reuters, New York, NY, 2018) software was used. After removal of duplicates, 146 publications were selected for manual screening. Two independent reviewers (E.W. and T.H.) subsequently assessed articles for eligibility; screening all articles by title and abstract first, and by full text thereafter. Three senior authors (M.G, T.M., and J.S.) were available for potential disagreements.

This scoping review was conducted according to the PRISMA (Preferred Reporting Items for Systematic Reviews and Meta-Analyses) Extension for Scoping Reviews (Tricco et al., 2018).

### 2.2 Eligibility criteria and study selection

We included original research articles in English or German reporting on ethical issues in neurosurgery. Topics included adults and children as well as cranial and spinal cases. Narratives, opinions, comments on original research articles and grey paper were excluded, as well as other literature reviews and case reports. Studies assessing different professions mixed with neurosurgery, where the neurosurgical part could not be clearly distinguished or represented only a small percentage, were excluded.

### 2.3 Data extraction and analysis

We reported descriptively on the study characteristics, country of origin (defined as the affiliated country of the first author), publishing year, topic of the study, study design and summarized the main outcomes. The topic and the main outcome are reported as perceived by the respective authors or, if not clearly indicated, as perceived by the reviewing team. We categorized the studies into six ethical subcategories from a neurosurgical point of view including Pediatric, Decision Making, Functional Neurosurgery, Developing Countries, Patient Care, and Artificial Intelligence (Fig. 2). In addition, the studies were categorized based on the three pillars of ethics – normative, professional, and sociological ethics (Table 1).

**Table 1:** Study characteristics.

Study	Study Type	Ethical Topic	Research design/Target group	Conclusion
gallo et al. (Gallo, 1985), 1985 USA	Qualitative	*Pediatric; Professional Ethics* Pediatric neurosurgeons' perspective on prevalence and purpose of hospital ethic committees (HEC)	Questionnaire sent to 117 pediatric neurosurgeons in the USA and Canada, 9 respondents in Canada representing 6 institutions; 85 respondents in the US, representing 74 institutions	• Increasing formation of HEC • Committees dominated by health professionals • No perceived impact of presence of HEC on frequency of court involvement → Ethical beliefs and attitudes towards ethics of pediatric Neurosurgeons play a key role in future success of HECs
gallo et al. (Gallo, 1991), 1991 USA	Qualitative	*Pediatric, Normative Ethics* Pediatric neurosurgeons' perspective on prevalence and purpose of hospital ethic committees	Questionnaire send to 158 pediatric neurosurgeons in the USA, Canada and Puerto Rico. 113 respondents, representing 93 institutions in the US and one in Puerto Rico, Canadian data not included in this report. Comparison to the first study by the same author	• Comparison to study from 1985 • HEC playing an increasing role, more participation of HEC in terminating life support and/or withdrawal of therapy • still dominated by physicians and nurses but gradually broadening profiles **→ End of life decisions in pediatric patients represent a delicate topic where an increasing support through HECs is utilized**
martin et al. (Martin et al., 2010), 2010 USA	Quantitative	*Pediatric, Sociological Ethics* Care of pediatric neurosurgical patients in Iraq in 2007 (war-related injuries and disease/nonbattle injuries)	Retrospective analysis of a prospectively maintained database. Exploring ethical challenges of the deployed environment. All consultations of the neurosurgical component of US Army 04–09/2007 in Iraq. 287 consultations of which 77 were Iraqi civilians	• 42/77 (15 %) patients were civilian Iraqi children, hereof 12 % with disease/nonbattle injury • overall, 67 % required surgical treatment • 19 % of the treated children died **→ Importance of appropriate triage of patients and ethical preparation for deployment**
rydvall et al. (Rydvall et al., 2007), 2007 Sweden	Qualitative	*Decision making, Sociological Ethics* Decision making in life-threatening cerebral condition (for and against life-sustaining treatment)	Questionnaire send to 298 ICU physicians and 112 neurosurgeons in Sweden. The response rate was 62.5 % for neurosurgeons and 73.5 % for ICU physicians.	• More neurosurgeons emphasized quality-of-life aspects • more ICU physicians considered patients previously expressed wish important • With worsening prognosis, judgment of the two groups converged **→ Differences in opinions seem to be based on divergent judgements of empirical facts, rather than basic moral values**
sobhani et al. (Sobhani et al., 2016), 2016 Iran	Qualitative	*Decision making, Sociological Ethics* Ethical theories used by neurosurgery resident to make decisions in challenging cases of medical ethics	Semi-structured interviews of 12 Iranian residents (all male and Muslim; 6 senior, 6 junior residents) regarding five common cases. Comparison of the dominant ethical theory used in each case	• In 50 % Deontology, in 50 % Consequentialism used, in two cases additionally virtue ethics. No one used a single ethical theory in all cases • No difference between junior or senior residents **→ Decision making dominated by deontology and consequentialism, ethical decisions seem not based on a single ethical theory but rather case-based**
mathies-en et al. (Mathiesen et al., 2022), 2022 DENMARK/SWEden	Qualitative	*Decision making, Sociological Ethics* Differences of ethico-legal practice between European countries	Questionnaire constructed to evaluate the use of ethical guidelines, consisting of 5 sections. First, reply by one senior consultant each of 29 European countries. Secondly, validation through representatives of each nation. Study conducted 2006–2007	• EANS/WFNS ethics code and good practice guideline largely unknown. • Views concerning life and death issues, the need to establish priorities and use of litigation differed extensively between the countries **→ Professional ethics and decisions making seems to be rather based on sociological and personal values than supported through ethical guidelines**
van der straeten et al. (Tricco et al., 2018), 2022 belgium	Qualitative	*Decision making, Normative Ethics* Ethical attitude in neurosurgery during the COVID-19 pandemic	Questionnaire including case vignettes distributed via the EANS mailing list (estimated amount of members 1500) and answered by 115 neurosurgeons, hereof 29 were trainees	• Agreement that fast-evolving oncology disease constitute an essential procedure • Rather unacceptable to withdraw treatment already stated as indicated **→ Healthcare crisis with resource limitations results in a standoff between patient care and preservation of resources for future patients, leading to ethical considerations of fairness**
Bell et al. (Bell et al., 2011), 2011 canada	Qualitative	*Functional, Sociological Ethics* Health care providers perspective on ethical and social challenges encountered in DBS	Study-invitation distributed via E-mail to health care providers working in Canadian DBS neurosurgery program. 20 health care of five different sites participated in the semi structured interview (not determinable how many individuals were invited but declined), content analysis of the interviews	Key ethical challenges included process of patient selection, resource allocation and long-term management **→ Ethical challenges regarding DBS seem to be of rather professional nature e.g. regarding infrastructure and communication in multidisciplinary teams, than normative ethical considerations**
ankeambom et al. (Ankeambom et al., 2021), 2021 cameroon	Quantitative	*Developing County, Sociological Ethics* Assessment of ethical dimensions of neurosurgical care in Cameroon	Two questionnaires answered by 77 patients (response rate 31,8 %) and 20 healthcare providers (response rate 71,4 %) of two Cameroonian neurosurgery centres between 11/2020 and 03/2021	• Both, health care providers and patients, reported lack of resources and infrastructure as greatest barrier. • 54 % of the patients spent 80–99 % of their annual household income on neurosurgical care **→ Ethical issues in low resource setting require different approaches and measures to improve ethical neurosurgical decisions making**
hughes et al. (Hughes, 2022), 2022 usa	Qualitative	*Developing Country, Sociological Ethics* Ethical challenges in the decision making of Ugandan neurosurgical care providers	In-depth structure interviews of 14 doctors providing direct neurosurgical care at the two largest hospitals in Uganda (35 individuals were expected to meet inclusion criteria, hereof at least one third was planned to be included)	Three ethical challenges that influenced the participants options and treatment choices were formal policies, surgical decision making, and resource limitations **→ Ethical beliefs and opinion regarding neurosurgical decision making in developing countries seem strongly influenced by external circumstances rather than normative considerations**
holmgren et al. (Holmgren, 2023), 2023 SWEden	Qualitative	*Patient care, Professional Ethics* Ethical and clinical aspects of restraint in neurosurgical care in Sweden	Mixed method design with four study-phases. Phase I: Study-specific questionnaire for neurosurgical patients (N = 58) and medical records. Phase II & III: Semi-structured interviews for neurosurgical nurses (N = 15). Phase IV: empirical findings, restraint regulations	• Restraint used for protection of patient/others, but also for convenience. • Certain patient characteristics associated with higher risk of restraint. • Nurses reasoning based on consequentialism **→ To cause the least suffering (For the patients or his surroundings) as anticipated by nurses in context of consequentialism seems to be a main goal of restraint**
lee et al. (Lee et al., 2024), 2024 south korea	Qualitative	*Artificial Intelligence, Professional Ethics* Artificial intelligence answers to medico-ethical questions in neurosurgery	Five multiple choice (MC) questions and 2 situations requiring ethical decision provided by the ethical committee. Uploaded to ChatGPT, Bing Chat and Googles Bard (in 11/2023), evaluation of the answers (comparison to the correct answers provided by the ethic committee)	• ChatGPT and Bard responded correctly to all 5 MC-questions, Bing Chat to only 3. • In the 2 ethical situations, ChatGPT presented answers that avoided ethical conflict, Bing Chat and Bard presented opinions based on their own judgment **→ The use of AI for ethical dilemmas poses an ethical challenge itself and should always be reviewed with human ethical considerations**
shlobin et al. (Shlobin et al., 2024), 2024 usa	Qualitative	*Artificial intelligence, Professional Ethics* Incorporation of Artificial intelligence (AI) into Neurosurgery	Six chatbots were asked to answer the questions how AI can be ethically incorporated in neurosurgery (12/2023). In Chatbots where the conversation style could be specified, the “precises” option was selected. Summary of the results and pareto analyse (with 20 % and 10 % threshold)	• Twelve key ethical considerations focusing on stakeholders, clinical implementation and governance were identified. • Pareto analysis top 20 % identified 10 key points, top 10 % identified 5 key points. **→ AI proposed 12 key topics where itself could support ethical considerations in neurosurgery. These need to be further explored in the future**

HEC = Hospital ethic committee, DBS = deep brain stimulation, ICU = intensive care unit, MC = multiple-choice, AI = Artificial intelligence

## 3 Results

**Fig. 1:**
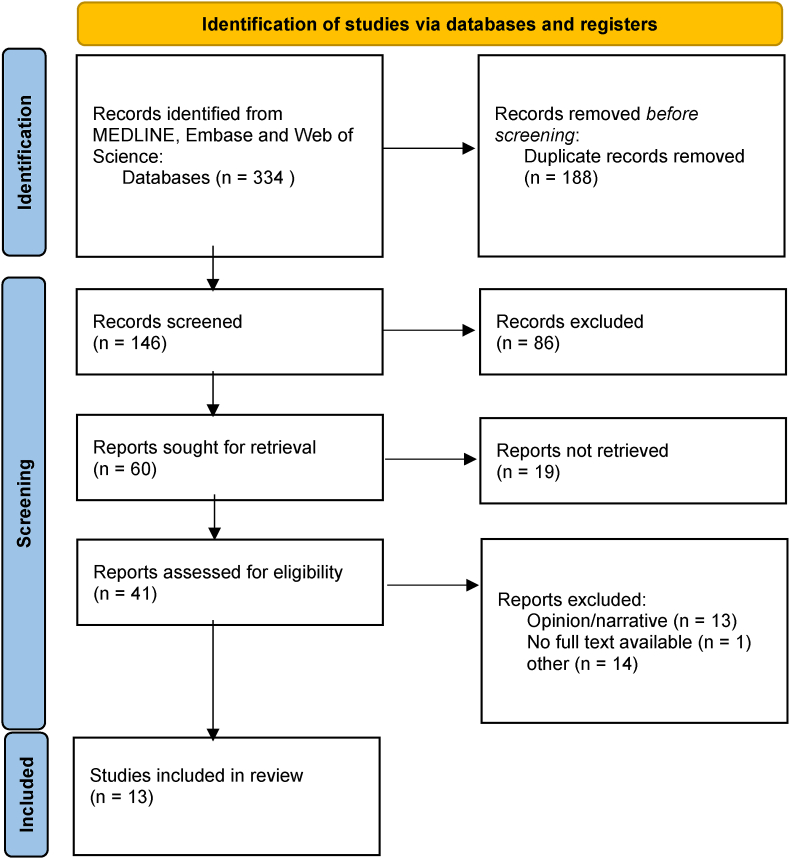
Flow-Chart for article inclusion.

We identified 334 records, of which 146 were screened and 41 retrieved for full text screening. The main reason for exclusion during full text screening, was the nature of the study being an opinion or commentary rather than original research. We identified 13 studies that were eligible for inclusion in this scoping review (Fig. 1, Table 1).

**Fig. 2:**
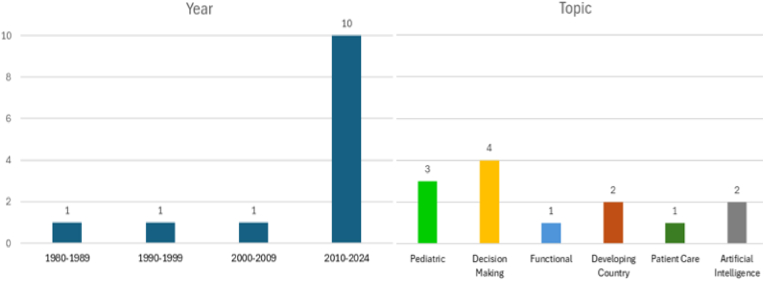
Distribution of publishing year and sub-categories.

**Fig. 3:**
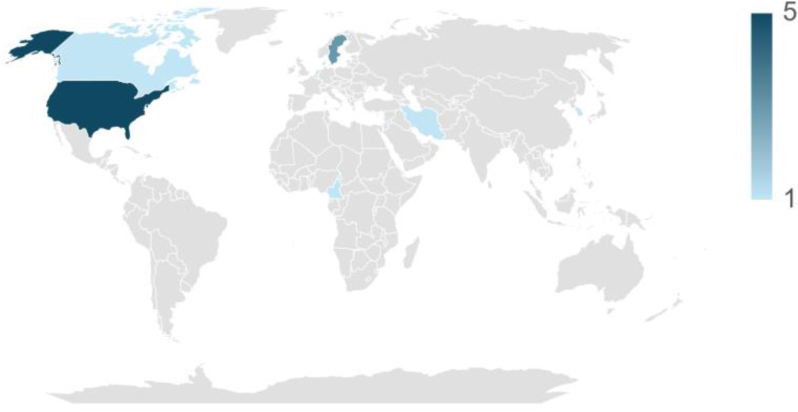
Map with the countries of study-origin.

The 13 studies included were published between the years 1985 and 2024. Ten (77 %) studies were published after 2010 and only three (23 %) between 1985 and 2009, as shown in Fig. 2. Six (46 %) studies originated in North America (five from USA, one from Canda), 4 (31 %) in Europe (two from Sweden, one from Sweden/Denmark, one from Belgium), and 1 (8 %) in Iran, Cameroon, and South Korea each (see Fig. 3).

All studies employed a qualitative research design. From an ethical standpoint seven studies focused on psychosocial ethical issues (53.8 %), four examined normative ethical questions (30.7 %), and two addressed aspects of professional ethics (15.4 %).

Four (31 %) studies discussed decision making as their main topic including decision making in life-threatening situations, ethical neurosurgical decision making during the Covid-19 pandemic, ethical theories used by neurosurgical residents, and differences of ethical decision making between European countries (Sobhani et al., 2016; Mathiesen et al., 2022; Rydvall et al., 2007; Van der Straeten et al., 2022). They demonstrated that overall neurosurgeons agree over some ethical questions and use similar ethical theories for decision making, nevertheless views between countries on other ethical topics can differ profoundly.

Three (23 %) studies discussed pediatric issues. Gallo et al. performed a survey evaluating the prevalence and purpose of hospital ethic committees (HEC) as viewed by pediatric neurosurgeons. Six years later, in 1991, they repeated the same survey with pediatric neurosurgeons to assess the evolution of HEC in this period (Gallo, 1985, 1991). These studies showed that over the years HEC involvement was increased in ethical decision makings, while the committees are still dominated by health professionals. Martin et al. investigated the care of pediatric patients in a US-army field hospital in Iraq, which we categorized into the pediatric sub-category but could also be categorized into developing countries (Martin et al., 2010). They emphasize the importance of appropriate triage of patients and ethical preparation for deployment.

Two (15 %) studies address issues in developing countries (Cameroon and Uganda) (Ankeambom et al., 2021; Hughes, 2022). Both studies concluded that lacking resources and infrastructure as well as high financial burden due to medical costs are the main ethical challenges in these countries. Two (15 %) studies, both published in 2024, investigated the incorporation of artificial intelligence (AI) in neurosurgery by asking different Chatbots the same questions (Lee et al., 2024; Shlobin et al., 2024). They demonstrated how Chatbots could be an auxiliar tool for medico-ethics issues but not replace human considerations at this point.

One study (8 %) discussed functional neurosurgery, inferring that key ethical challenges of deep brain stimulation include patient selection and long-term management (Sobhani et al., 2016). One study (8 %) discussed patient care, with the use of restraint in neurosurgical care, demonstrating how certain patient characteristics are associated with higher risk of restrain and how reasons for use of restraint can differ (Bell et al., 2011; Holmgren, 2023).

Overall, 10 studies (77 %) used questionnaires and/or (semi-) structured interviews as their study method. Martin et al. was the only group that solely analysed their database to assess how many children were treated at the US-Army field hospital in Iraq and for which pathology (Martin et al., 2010). Nine studies (69 %) directed their questions towards a cohort of neurosurgeons and/or neurosurgical care providers. Sobhani et al. questioned specifically neurosurgical residents, and Ankeambom et al. was the only group also questioning patients beside neurosurgical healthcare workers (Sobhani et al., 2016; Ankeambom et al., 2021). Lee et al. and Shlobin et al. did not directed their questions to humans, but to different Chatbots (Lee et al., 2024; Shlobin et al., 2024).

The main results of each study are presented in Table 1.

## 4 Discussion

The aim of this study was to provide a systematic overview on the topics and study designs used for research regarding ethical issues in neurosurgery. Ethical considerations in neurosurgery present a broad variety of topics and patient groups and are therefore difficult to quantify. Approximately half of the included studies have been conducted in North America, while the European studies were predominantly conducted in Northern Europe. Most included studies address a specific ethical issue while only three studies evaluated a more general perspective of ethical decisions in neurosurgery. Of the included studies, 92 % employed questionnaires or (semi-)structured interviews, with the vast majority published after 2010. This trend likely reflects increased awareness of the importance of ethical considerations in neurosurgery, as well as growing efforts to enhance the quality of ethical decision-making through structured research. The topics dealt with in the included articles were ethics concerning pediatric patients, patient care, decision making processes, artificial intelligence, developing countries, and functional neurosurgery. From an ethical perspective, most articles focus on sociological aspects, aiming to understand how ethical norms are applied in practice across various neurosurgical subspecialties, professional groups, and clinical contexts.

The existence of only 13 studies addressing ethics in neurosurgery, with the majority published after 2010, underscores a significant research gap in original investigations of ethical issues within the field. While awareness of the importance of ethics in neurosurgery has increased since 2010, it remains generally limited overall. Many factors can influence ethical decision making, including, but not limited to, religion, ethnic background, technical innovation, and available resources (Hughes, 2022; Bernstein et al., 2004; Ibrahim et al., 2015). Therefore, it can be very challenging to consider all factors when discussing ethical decision-making (Stengel et al., 2024, 2025) and conducting ethical research. Interestingly, we did not identify any publications that specifically evaluated the impact of religious or cultural backgrounds on ethical decision-making. Nevertheless, clinical experience suggests that these factors frequently play a significant role in everyday decision-making processes. Religious beliefs and personal worldviews can shape moral values, perceptions of suffering, and attitudes toward life-sustaining treatments, thereby influencing how ethical dilemmas are interpreted and resolved. Awareness of these influences is therefore essential for ethically sensitive and patient-centred clinical practice.

Comprehensive research as well as the implementation of guidelines for ethical considerations, decisions, and discussions should be sought and would potentially simplify ethical decision-making for health-care workers and even patients and their families.

Some of the papers included in this review do not address ethical issues from a purely philosophical standpoint but rather focus on what the authors themselves perceive as ethical discussions. For example, Martin et al. examined neurosurgical care for war-related and non-war-related pediatric patients in Iraq. While providing healthcare—particularly to children—in war zones undoubtedly raises profound ethical dilemmas, the study primarily reported quantitative data, such as the number of children treated and the types of conditions addressed, rather than engaging with ethical questions surrounding the treatment of 'enemy’ patients in conflict settings (Martin et al., 2010). Similarly, Ankeambom et al. sought to identify factors influencing ethical decision-making in Cameroon. However, the study did not clearly define what constituted ethical decision-making and instead concentrated on infrastructural barriers to optimal neurosurgical care from a medical standpoint (Ankeambom et al., 2021). These examples illustrate that ethical considerations as viewed by neurosurgeons in practice may diverge significantly from traditional philosophical approaches to ethics and may be shaped more by contextual and practical challenges than abstract moral theory. When examining the arguments used by neurosurgeons and intensive care unit physicians both in favor of and against life-sustaining treatments for critically ill patients, quality-of-life considerations emerged as a central factor in both groups (Rydvall et al., 2007). However, neurosurgeons were significantly more likely than ICU physicians to identify quality of life as the most important argument. In contrast, only a minority in both groups considered the patient's wish to avoid a persistent vegetative state to be a key factor, although ICU physicians significantly more often ranked this consideration as the most important. While there is broad agreement between neurosurgeons and ICU physicians regarding initial treatment decisions—such as proceeding with life-saving surgery in critically ill patients—differences tend to diminish as the clinical course progresses and further decisions must be made. In later stages, including decisions about withdrawal of mechanical ventilation due to extensive brain damage or the administration of opioids or sedatives despite the potential to hasten death, the perspectives of both groups become increasingly aligned. This convergence suggests that observed differences in opinion may be driven primarily by divergent assessments of empirical facts, such as prognosis and expected outcomes, rather than by fundamental ethical or moral values.

One of the most dominant ethical issues in neurosurgery, is end-of-life decision-making in critical or terminally ill patients (Hautmann et al., 2024). Surprisingly, only one publication studied this topic (Rydvall et al., 2007). Rydvall et al. discovered, that neurosurgeons placed greater emphasis on the quality of life in these patients compared to intensive care unit (ICU) physicians (Rydvall et al., 2007). On the other hand, more ICU doctors emphasized the importance of respecting the patients previously expressed will. Both views can be critically examined within an ethical framework. It may be argued that there is no universally accepted definition of good quality of life, making assessments inherently subjective. Conversely, patients may face challenges in making advanced decisions about managing critical situations, as they have likely never experienced such circumstances firsthand. An additional important component of end-of-life decision-making concerns the withholding or withdrawal of care in general, and neurosurgical interventions in particular. Similar ethical principles apply to both withholding and withdrawing treatment, as both involve deliberate decisions to limit life-sustaining interventions. Despite the fact that such decisions constitute a substantial and routine part of neurosurgical practice, the literature addressing this topic remains limited. Rydvall et al. sought to examine the factors influencing neurosurgeons’ decisions to withhold treatment, withdraw life-sustaining therapy, or administer medications that may hasten death. However, owing to the complexity, context dependence, and highly individualized nature of these decisions, no definitive decision-making framework or generalizable guidelines could be derived from their findings (Rydvall et al., 2007). This example highlights the inherent subjectivity of ethical decision-making and individual autonomy in medical ethics.

The two most recent studies from 2024 examined the integration of AI into neurosurgical practice. Over the past year, AI has been a topic of increasing relevance and debate, not only in the medical sector, but across various fields. While these emerging technologies offer the potential to enhance clinical workflows, concerns have been raised regarding their reliability and implementation, particularly in a sensitive domain such as healthcare (Mishra et al., 2023). The included studies indicated that AI models, such as ChatGPT, can contribute to ethical discussions and may serve as a supportive tool for both neurosurgeons and patients. However, current AI systems are not yet validated for this application and exhibit notable limitations (Stengel et al., 2024, 2025). Consequently, their use should be approached with caution, functioning as an auxiliar tool rather than a replacement for human ethical judgment.

The two earliest studies examined the role of HEC in neurosurgical decision-making. In the 1980s and 1990s, ethic committees were not widely established in many hospitals; however, over time HECs and ethical rounds have become an integral part of numerous medical departments (Ford et al., 2006; Schmitz et al., 2018). This evolution demonstrates how certain ethical considerations transition into routine clinical practice, while emerging innovations continue to introduce new ethical challenges and considerations.

Guidelines, as those provided by the World Federation of Neurosurgical Societies, can serve as valuable support in addressing ethical challenges in clinical practice (Umansky et al., 2011). However, awareness of these guidelines among neurosurgeons appears to be limited, raising question about the extent to which ethical consideration is actively integrated into routine clinical decision-making (Mathiesen et al., 2022). Extraordinary circumstances, such as war or the Covid-19 pandemic, often prompt a re-evaluation of ethical principles and intensifies ethical debates (Ozisik et al., 2022; Martin et al., 2010; Tsermoulas et al., 2020). However, ethics education and its practical application should not be limited to crisis situations but should be systematically incorporated into neurosurgical training (Balak et al., 2022). While many surgeons develop their own ethical framework over time, enhanced ethics education could better equipe young doctors to navigate complex ethical dilemmas in clinical practice.

## 5 Future research directions

One of the main challenges in researching ethics in healthcare is the lack of validated measurement tools. As our review indicates, most studies rely on customized questionnaires or (semi-) structured interviews, making direct comparison difficult. Only one study collected retrospective patient data, but it lacked clear documentation of ethical considerations underlying decision-making (Martin et al., 2010). To obtain comparable and prospective data, particularly on ethical decision-making, future research should prioritize the development of validated questionnaires for assessing ethical management. In addition, future research on ethical dilemmas in neurosurgery should be more explicitly grounded in the core philosophical pillars of ethics—normative, professional, and sociological ethics —to ensure a deeper and more systematic exploration of moral challenges. Integrating these frameworks can help bridge the gap between practical concerns and foundational ethical reasoning. Furthermore, evidence suggests, that only a small number of residents receive formal education in medical ethics (Sobhani et al., 2016; Rajmohamed et al., 2017). Nevertheless, continuous ethical education is essential to ensure competent medical practice (Balak et al., 2022; Tisell et al., 2020). Therefore, we advocate for further development and reinforcement of ethical education within neurosurgical residency programs. In addition, this may be achieved through targeted educational initiatives within neurosurgical societies (e.g., the EANS), including ethical aspects within the framework of training courses for residents and early-career consultants. Such programs should incorporate expert-led teaching on fundamental ethical principles, complemented by discussions of neurosurgery-specific ethical challenges and, where feasible, providing trainees with recommendations or guidelines addressing particular ethical issues in neurosurgical practice.

Ethical considerations in a global aspect must account for diverse cultural, religious, and societal factors. This is particularly relevant in end-of-life decisions, where perspectives can vary significantly. When developing ethical training programs or guidelines, it is essential to incorporate these differences. While future research should also specifically examine how cultural and religious backgrounds influence ethical decision-making in clinical practice across different regions of the world. Comparative, cross-regional studies would be particularly valuable to identify similarities and differences in ethical reasoning, thereby informing more culturally sensitive guidelines and global neurosurgical practice. Additionally, research focusing on the ethical aspects during long term follow up after, for example, neurosurgical intervention in severe brain injuries or other critically ill patients is still needed (Honeybul, 2018). Expanding HECs to include a multidisciplinary team not only healthcare professionals but also individuals with cultural religious expertise could enhance the depth of ethical deliberations (Gallo, 1991; Ford et al., 2006). The ethical guidelines established by the EANS in 1999 provide a valuable framework for neurosurgeons and should be applied flexible to accommodate different clinical situations (World federation of neurosurgical societies, 1999). However, studies such as from the group of Mathiesen et al. indicate that most European neurosurgeons are unaware of these guidelines (Mathiesen et al., 2022). To increase awareness, we recommend actively promoting ethics discourse, for example, by dedicating sessions to ethics at the annual neurosurgical congresses. Given that these guidelines were established over two decades ago, they should be updated to reflect contemporary challenges and advancements. Our review highlights that recent studies have increasingly focused on integration of AI into neurosurgical ethics. However, these emerging technologies have yet to be incorporated into existing ethical guidelines or formal education. Future research should address this gap and explore how AI can be ethically implemented in neurosurgical practice. The recommendations are summarized in Table 2.

**Table 2:** Recommendations for future research and implementation of ethics in Neurosurgery.

Category	Key Recommendations
**Research Methods & Measurement**	Develop validated, prospective questionnaires for ethical decision-making to improve comparability and rigor
**Ethical Frameworks**	Ground future research more explicitly in normative, professional, and sociological ethics to strengthen theoretical foundations and link practice with moral reasoning
**Education & Training**	Expand and reinforce formal ethics education in neurosurgical residency through EANS courses, lectures, and inclusion of ethics in board examinations
**Cultural & Global Perspectives**	Incorporate cultural, religious, and societal differences into ethical training, guidelines, and end-of-life decision-making frameworks
**Ethics Committees (HECs)**	Strengthen HECs by including multidisciplinary members, such as cultural and religious experts, to enhance ethical deliberation
**Guidelines & Awareness**	Increase awareness of EANS ethical guidelines through active promotion (e.g., congress sessions) and update them to reflect modern challenges
**Emerging Technologies (AI)**	Investigate ethical implications of AI in neurosurgery and integrate these considerations into updated guidelines and formal education

## 6 Limitations

This scoping review is subject to the inherent limitations associated with this type of research. Our searched was restricted to publications with the specified MeSH terms in the title, which may have led to the possible omission of relevant articles. For instance we recognise that our search strategy may have omitted ethical studies that are not exclusively neurosurgical although relevant to our practice (e.g. value or life, autonomy, etc.). Furthermore, as previously mentioned, an abstract-based search resulted in an overwhelming number of publications due to the widespread use of the term “ethics” across various contexts. To ensure a more focused and relevant dataset, a “title-only” search strategy was ultimately selected. Another limitation is that the inherent variability in the interpretation of the term “ethics”. We had to rely on the authors’ appropriate use of the term within their research, which introduces potential inconsistencies. Ultimately, what different authors classify as an “ethical” issue may vary, affecting the scope and comparability of the included studies. Almost all included studies employed interviews and/or study-specific questionnaires as their primary data collection method. While this approach is reasonable given the inherent challenges in quantifying ethical issues, it results in limited comparability between qualitative studies and, consequently, weak evidence for informing decision-making processes.

## 7 Conclusion

While “ethics in neurosurgery” is a broad topic relevant to most health-care providers in daily practice, it remains underrepresented in current research. There is a growing trend towards discussion on ethical challenges arising from emerging technologies, such as AI. Additionally, exceptional circumstances, such as wars and pandemics, have been shown to intensify ethical debates and prompt re-evaluations of ethical principles. However, there is a notable lack of validated study designs or standardized methods for assessing ethical issues in research, while ethical dilemmas in neurosurgery, as defined by neurosurgeons, often do not align with the core philosophical pillars of ethics, highlighting a gap between practical perceptions and foundational ethical theory. Lastly, the limitations of this scoping review do not degrade our conclusions, which are also valid in terms of lack of research/application of ethics in neurosurgery education, an aspect that holds paramount importance for the junior generations of neurosurgeons and neurosurgical trainees. Engaging into this conversation continue to represent a priority for the EANS and its Ethico-Legal Committee, and we formally commit to improve the *status quo* through renewed projects for the years to come.

## Conflict of interest

The authors have no competing interests to disclose.
